# Mechano growth factor, a splice variant of IGF-1, promotes neurogenesis in the aging mouse brain

**DOI:** 10.1186/s13041-017-0304-0

**Published:** 2017-07-07

**Authors:** Jason J. Tang, Jewel L. Podratz, Miranda Lange, Heidi J. Scrable, Mi-Hyeon Jang, Anthony J. Windebank

**Affiliations:** 1 Department of Neurology, Mayo Clinic College of Medicine, 200 First Street SW, Rochester, MN 55905 USA; 2 Department of Laboratory Medicine and Pathology, Mayo Clinic College of Medicine, Rochester, MN USA; 3 The Kogod Center on Aging, Mayo Clinic College of Medicine, Rochester, MN USA; 4 Department of Neurologic Surgery, Mayo Clinic College of Medicine, Rochester, MN USA

**Keywords:** Mechano growth factor (MGF), Neural stem cells, Neurogenesis, Insulin-like growth factor-I (IGF-I), Neuroprotection, Aging

## Abstract

Mechano growth factor (MGF) is a splice variant of IGF-1 first described in skeletal muscle. MGF induces muscle cell proliferation in response to muscle stress and injury. In control mice we found endogenous expression of MGF in neurogenic areas of the brain and these levels declined with age. To better understand the role of MGF in the brain, we used transgenic mice that constitutively overexpressed MGF from birth. MGF overexpression significantly increased the number of BrdU+ proliferative cells in the dentate gyrus (DG) of the hippocampus and subventricular zone (SVG). Although MGF overexpression increased the overall rate of adult hippocampal neurogenesis at the proliferation stage it did not alter the distribution of neurons at post-mitotic maturation stages. We then used the *lac*-operon system to conditionally overexpress MGF in the mouse brain beginning at 1, 3 and 12 months with histological and behavioral observation at 24 months of age. With conditional overexpression there was an increase of BrdU+ proliferating cells and BrdU+ differentiated mature neurons in the olfactory bulbs at 24 months when overexpression was induced from 1 and 3 months of age but not when started at 12 months. This was associated with preserved olfactory function. In vitro, MGF increased the size and number of neurospheres harvested from SVZ-derived neural stem cells (NSCs). These findings indicate that MGF overexpression increases the number of neural progenitor cells and promotes neurogenesis but does not alter the distribution of adult newborn neurons at post-mitotic stages. Maintaining youthful levels of MGF may be important in reversing age-related neuronal loss and brain dysfunction.

**Electronic supplementary material:**

The online version of this article (doi:10.1186/s13041-017-0304-0) contains supplementary material, which is available to authorized users.

## Introduction

Neurogenesis occurs in two major regions of the adult brain, the subventricular zone (SVZ) of the lateral ventricles and the subgranular zone (SGZ) of the hippocampus [1–4]. The process of neurogenesis in the adult mammalian brain appears to be a dynamic process that may be influenced by factors, such as age, exercise, stress and disease states [5, 6]. Stem cells in the neurogenic regions decline with age, but do not disappear altogether [7]. Loss of neurons is associated with decreased function in regions replenished by adult neurogenesis. Maintenance of neural stem cell (NSC) populations appears to be critical for optimal function of the brain. One potential approach to conserving brain function in the aging brain would be to maintain NSC populations and adult neurogenesis at youthful levels.

Mechano growth factor (MGF) is a unique, spliced variant of insulin-like growth factor 1 (IGF-1). The IGF-1 gene includes 6 exons and generates 5 distinct mRNAs coding for three distinct isoforms: IGF-1Ea, IGF-1Eb and IGF-1Ec through alternative mRNA splicing [8, 9]. IGF-1 is found in various tissues in the body including liver and muscle. The liver isoform of IGF-IEa is different from the muscle IGF-I isoforms (IGF-1Eb, IGF1-Ec).

When muscle tissues are stretched or mechanically damaged the level of IGF-1Eb mRNA expression (in rodent) or IGF-1Ec mRNA expression (in human) increases. Because these increases were identified after mechanical damage and expressed in a mechano-sensitive manner, IGF-IEb mRNA was named mechano-growth factor (MGF) [10, 11].

MGF has been shown to stimulate muscle stem cells (satellite cells) to re-enter cell cycle and proliferate, resulting in new muscle cells to replace injured fibers [10, 12]. A similar role of MGF has been explored in chondrogenesis [13] and differentiation of mesenchymal stromal cells [14]. Recently MGF has been shown to have neuroprotective effects in an ischemic brain model [15] and it had been reported to be markedly more effective than IGF-I [16].

Although these studies showed a protective role for MGF, the function of MGF is still not well understood. Using a novel, rabbit-derived polyclonal antibody specific for IGF-1Eb, we demonstrate for the first time the specific patterns of neuronal expression of MGF in mouse brain and that MGF plays a role in preventing neuronal attrition and brain dysfunction in aging mice. Our studies utilized mice that constitutively or conditionally overexpress MGF in the brain.

## Methods

### Animals

All animal studies were performed in compliance with Association for Assessment and Accreditation of Laboratory Animal Care International and under the supervision of the Mayo Clinic Institutional Animal Care and Use Committee. All mice were housed in a facility with a 12-h light ⁄ dark cycle with free access to food and water. A total number of 60 animals were used in this study.

**Fig. 1:**
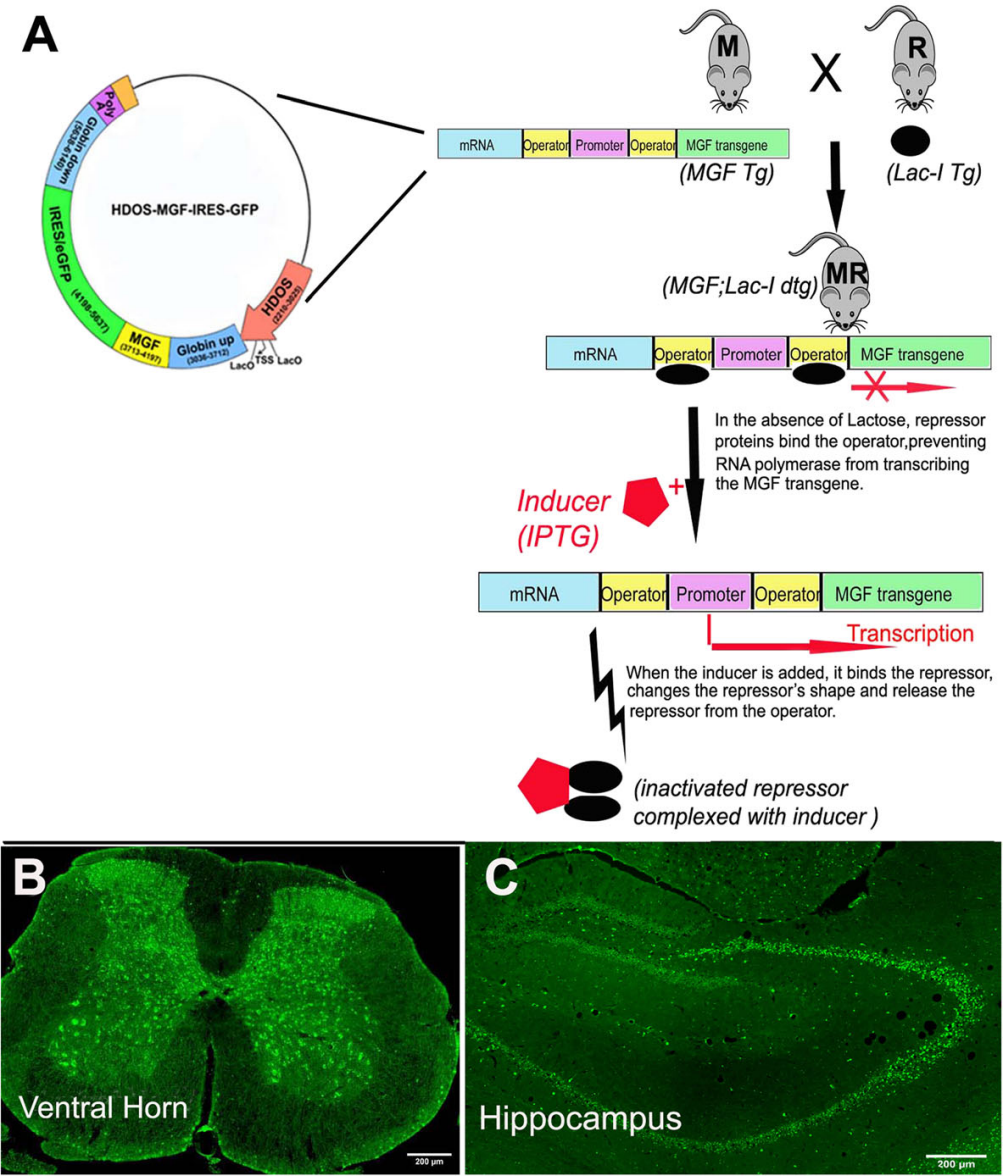
**a** Scheme for generating the inducible overexpression of MGF based on the *Lac* operon system. Isopropyl β-D-1-thiogalactopyranoside (IPTG) is a molecular mimic of allolactose, a lactosemetabolite that triggers transcription of the *Lac* operon. We generated a new inducible transgene utilizing the promoter from the Huntington’s disease (HD) locus that was modified to include 2 *Lac* operators flanking the transcription start site to drive expression of the MGF-EGFP-Luc chimeric cDNA. When the mouse is fed with IPTG dissolved in daily water, the *Lac* operon is de-repressed and MGF is transcribed. The HDOS promoter drives expression specifically in neurons. GFP expression, which represents the MGF transgene, was restricted to neurons of selected area including (**b**) spinal cord and (**c**) hippocampus (negative controls not shown)

Two transgenic mouse lines were established using the *lac*-operon system, the HD^LacO^-MGF transgenic mouse (referred as M mice) and Lac I transgenic mouse (referred as R mice) (Fig. 1a). In M mice, MGF was expressed using the HD locus promoter which was modified to include *lac* operators and in R mice, the human β-actin promoter was used to drive the Lac-I gene expression. Both transgene constructions have been previously utilized in our studies [17, 18] when overexpressed MGF was identified in spinal cord and hippocampal neurons (Fig. 1b and c).

### MGF antibody

The MGF polyclonal antibody was generated in collaboration with EMD Millipore Corporation. The mouse 2.94 kDa MGF peptide (SPSLSTNKKTKL-QRRRKGSTFEEHK) was used to immunize rabbits and generate antibodies. This antibody has subsequently been commercialized by EMD Millipore, Billerica, MA (UniProt Number P05017).

### Tissue preparation and immunohistochemistry

Mice were euthanized by intraperitoneal injection of pentobarbital. The brains were fixed with 4% paraformaldehyde (PFA) in phosphate buffered saline (PBS) at 4 °C for 24 h and cryopreserved with 30% sucrose. Serial frozen coronal sections through the OB, the RMS and SVZ were cut at 25 μm thickness using a Leica CM1850 cryostat (Leica Microsystems, Nussloch, Germany). For immunostaining, floating sections were blocked with 10% normal goat serum (NGS), 3% bovine serum albumin (BSA), 0.2%Triton X-100 in PBS at room temperature (RT) for 1 h. Sections were then incubated at 4 °C for 24–48 h with the following antibodies diluted in PBS supplemented with 10% NGS and 3% BSA. Rabbit anti-MGF (1:1000, Millipore, Billerica, MA), mouse anti-GFAP (1:200, MAB360, Millipore, Billerica, MA), guinea pig anti-DCX (1:300, AB2253, Millipore, Billerica, MA), mouse anti-Nestin (1:500, MAB353, Millipore, Billerica, MA), rabbit anti-NeuN (1:500,12943S, Cell Signaling Technology, Beverly, MA). For BrdU immunostaining, floating sections were incubated in 50% formamide, 2× SSC at 65 °C for 2 h and then treated with 2 M HCl at 37 °C for 30 min. After being incubated in 0.1 M Na borate at RT for 30 min, sections were blocked as described above and incubated at 4 °C for 48 h with mouse anti-BrdU (1:1000; B8434 Sigma, St. Louis, MO). Sections were incubated with Alexa Fluor goat anti-rabbit IgG and Alexa Fluor goat anti-mouse IgG (1:500, Life Technologies, Grand Island, NY) at RT for 1 h and counterstained with DAPI. Images were taken using Zeiss LSM 780 Laser Scanning and Zeiss Axiophot fluorescent microscopes.

### Bromodeoxyuridine (BrdU) injections

BrdU (Sigma, St. Louis, MO) was dissolved in 0.9% saline containing 0.007 M NaOH at a concentration of 5 mg/ml. To determine if MGF co-localizes with BrdU positive proliferating cells in the SVZ, mice received two treatments of BrdU (100 mg/kg; i.p.) 24 h apart through intraperitoneal (IP) injection and were sacrificed 2 h after the second injection. Mouse brains were prepared and immunostained as previously described.

### Neuronal differentiation and maturation assay

MGF overexpressing mice (M mice) and control (R mice), were injected with BrdU (50 mg/kg body weight, once daily at 10 a.m.) for 1 week and sacrificed 4 weeks after the first BrdU injection. Each group consisted of 4 mice. Coronal brain sections (40 mm thick) were prepared from injected mice and processed for immunostaining and confocal imaging. Stereological quantification within the SGZ and granule cell layer of the hippocampus was carried out as we previously described [19]. Briefly, BrdU labeling requires the following pretreatment: DNA denaturation (2 M HCl, 30 min at 37 °C). Primary antibody was a rat monoclonal anti-BrdU antibody (Novus Biologicals, NB500–169, 1:500; overnight incubation) LSM 780 confocal system (Carl Zeiss) with X40 objective lens and multi-track configuration. Stereological quantification of BrdU, DCX, NeuN cells was carried out. BrdU+/NeuN−/DCX+ cells were considered as newly generated immature neurons. BrdU+/NeuN+/DCX- cells were considered as newly generated mature neurons. BrdU+/NeuN+/DCX+ cells were considered as newly generated neurons in the transition of immature to mature phase. An observer blind to the genotypes and treatment of the animals performed assessments. Statistical significance was assessed by one-way ANOVA.

### Conditional MGF expression

M mice and R mice were bred together to produce a HD^LacO^-MGF; Lac-I double transgenic mouse (referred as MR mice) with conditional MGF expression. MGF transgene expression was induced by the lactose analog, Isopropyl β-D-1-thiogalactopyranoside (IPTG) (1758–1400, Inalco Pharmaceuticals, San Luis Obispo, CA). MR mice were fed 10 mM IPTG added daily to their drinking water beginning at 1, 3 and 12 months of age. The mice were kept until 24 months at which time behavioral, BrdU incorporation and histology assays were performed. Four littermate control groups were kept until 24 months of age. Control groups are as follows: (1) MR mice with normal water in which MGF remains repressed (controls for non-specific effects of the transgenic background); (2) M mice with normal water; (3) R mice with IPTG water (controls for the effects of IPTG); (4) R mice with normal drinking water (normal controls). We used 5 mice per group.

### Olfactory function analysis

Behavior analysis was performed at 24 months old in mice treated with and without IPTG. Briefly, all animals were familiarized with the odor of food for 2–3 days before testing and subjected to 18–24 h of food deprivation before the test day. The subject mouse was placed in a clean regular cage containing 3 cm of clean bedding and acclimated to the cage for 5 min. The mouse was then transferred to another empty, clean cage. The food target was buried approximately 1 cm beneath the surface in a corner of the first cage and the mouse was transferred back into the cage with buried food. The observer used a stopwatch to measure the time to find the buried food. If the subject failed to find the target after 15 min the test was stopped and 900 s recorded as the latency score. Statistical analysis was performed after function assay. Each animal was tested only once and 5 mice were used per group. Statistical analysis was performed using the student t-test.

### BrdU labeling of olfactory bulb for neurogenesis

MGF expression was induced in MR mice from 1, 3 and 12 months of age with IPTG. Prolonged (2 weeks) BrdU labeling was used to label both slower and rapidly dividing cells. At 24 months of age, MR and age matched R mice were given BrdU (1 mg/ml) dissolved in their daily water for 2 weeks. Mice were sacrificed 12 days after BrdU withdrawal. This was followed by immune-histological analysis as previously described. (*n* = 5 mice per group).

### Cell counting

After the functional outcome test, the numbers of BrdU+/NeuN+ mature neurons in the Olfactory Bulb (OB) were counted in 1 out of 10 consecutive, adjacent sections from the anterior portion of the OB to the beginning of the accessory OB. The number of double positive cells per slide was multiplied by 10 to estimate the total number of cells within the OB. The observer counting the neurons was blind to the experimental conditions. We counted 4–5 sections per mouse brain and 5 mice (20–25 sections total) were used per group. Statistical analyses were performed using SPSS (11.0). One or two-way ANOVA was performed as appropriate to determine statistical significance. A value of *p < 0.05 and ** *p* < 0.01 were considered to be statistically significant. All values are displayed as Mean ± SEM.

### Neurosphere culture

Published methods were followed [20–22]. Briefly, one mouse was selected from M, R and MR mice at 1, 3 and 24-month old. The brains were held on ice and coronally sectioned into 400 μm slices. Both sides of the SVZ area from each brain was isolated with fine forceps under the dissecting microscope, cut into small cubes (approximately 1.0 mm^3^) and 1 ml of 0.25% Trypsin-EDTA (Gibco, Carlsbad, CA) was added. Samples were incubated at 37 °C for 30 min during which time they were gently triturated through a Pasteur pipette every 10 min. After addition of 1 ml of trypsin inhibitor solution (Life Technologies, Grand Island, NY) the cell suspension was filtered through a hydrophilic PVDF filter unit (Pore Size 0.22 μm, Millipore, Billerica, MA). The filtered cell suspension was centrifuged for 5 min at 250 X g. The supernatant was removed, cells re-suspended in 1 ml of NSC medium (Gibco, Carlsbad, CA) and triturated to produce a single cell suspension. Viable cell concentration was measured using a hemacytometer and Trypan Blue exclusion dye. Dissociated cells were plated in 12-well plates at a density of 5 × 10^3^ cells/well in 0.5 ml DMEM containing N2 Supplement, B27 supplement, 20 ng/ml recombinant human EGF and 20 ng/ml recombinant human bFGF (all from Life Technologies, Grand Island, NY).

### Neurosphere proliferation assay

To determine the mitogenic effects of MGF, cultured neurospheres (NS) from MR mice were used to observe the effects of MGF on cell proliferation of SVZ-derived NSCs. R mice were used as background controls. MGF overexpression was induced by adding 5 mM IPTG to the culture medium. NS cultures were observed for the number and diameter of NS grown in the presence or absence of IPTG. The total number of neurospheres (NS) at passage 0 (P0) was determined at 7 days in vitro (7 DIV) by counting all NS generated from initial 5 × 10^3^ dissociated cells in each well. We quantified 12 wells per group. Phase contrast images of NS were captured at 7 DIV using a Zeiss inverted microscope equipped with a Sony DSC-S7 digital camera and a 10X objective. The diameter of NS was determined using ImageJ (developed by W.S. Rasband, NIH). We also used NS size as a measure of NSC proliferation as described [23]. Statistical analyses were performed using one-way ANOVA to determine statistical significance. A value of *p < 0.05 and ** *p* < 0.01 were considered to be statistically significant. All values are displayed as Mean ± SEM.

## Results

### Endogenous expression of MGF in selected areas of the brain

**Fig. 2:**
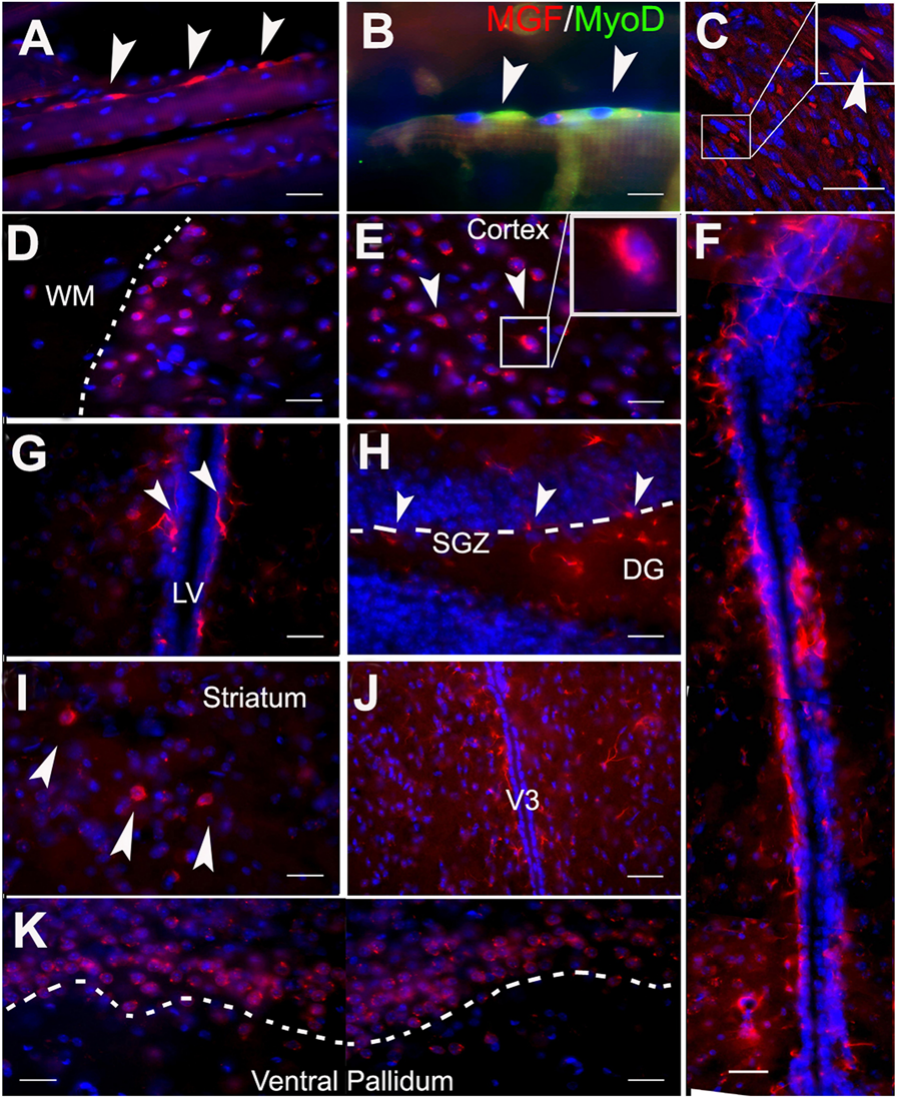
Identification and localization of MGF. **a** In the skeletal muscle, MGF-positive cells are distributed along the basal membrane of the muscle fiber. **b** MGF is also co-localized in MyoD-positive satellite cells along the basal membrane. Red is MGF, Green is MyoD). **c** MGF is expressed in the peri-nuclear region of heart muscle cells. **d** at the gray/white junction of the cerebral hemispheres (dotted line), MGF+ neurons were found within the cortical gray matter, rather than in white matter (WM). **e** in the cortex MGF-positive neurons were round with very short processes. **f** In the lateral ventricular (LV) region, MGF was distributed along the wall of LV. **g** under higher magnification, MGF-positive cells exhibited long branched cellular processes like astrocytes. **h** In the hippocampus MGF positive cells were distributed at the border of the of the subgranular zone (SGZ)and dentate gyrus (dotted line) which is an area of active adult neurogenesis. **i** A small number of rounded MGF positive neurons were also observed in the striatum. **j** In the hypothalamus cells were detected in the paraventricular (V3) area. **k** Numerous cells were also seen in ventral pallidum area of the basal ganglia. (*Red* is MGF, *Blue* is DAPI, Scale bar, (in A-K) 100 um)

MGF is expressed endogenously in skeletal and heart muscle. To test the specificity of our new MGF antibody, we confirmed the endogenous MGF expression in adult mouse skeletal muscle (Fig. 2a) and heart muscle (Fig. 2b and c). The observed distribution along the basal membrane of somatic muscle fibers and. co-localization with MyoD in heart muscle satellite is similar to that previously reported [24–26]. We then immunostained for endogenous MGF expression in various regions of the brain using wild-type 8–12 week old and 20 month old mice. There was wide-spread expression of MGF in neurons in younger mice that was not present in the aged mice (data not shown). In the cerebral hemispheres of the young mice, the majority of MGF-positive cells were located in the external granular layer of the cortex rather than in white matter (Fig. 2d). Positive cells had a round shape and co-stained with the nucleus (DAPI) (Fig. 2e). In the lateral ventricular (LV) region, MGF was distributed along the wall of LV (Fig 2f). MGF positive cells were also expressed in SVZ, which is a part of the brain where active adult neurogenesis occurs. Interestingly, under higher magnification, MGF-positive cells exhibited long branched cellular processes like astrocytes (Fig. 2g). In addition, MGF was also detected in the hippocampus (Fig. 2h), striatum (Fig. 2i), paraventricular (3rd Ventricle or V3) area of hypothalamus (Fig. 2j), and ventral pallidum (Fig. 2k). In the hippocampus, the positive cells were scattered in the subgranular zone (SGZ) of the dentate gyrus and extended their processes into the granular layer of the hippocampus (Fig 2h). MGF-positive cells were also seen within the hilus of the hippocampus and there was a high density of MGF stained cells in the ventral pallidum.

### Characterization of endogenous MGF-expressing cells in the adult brain

**Fig. 3:**
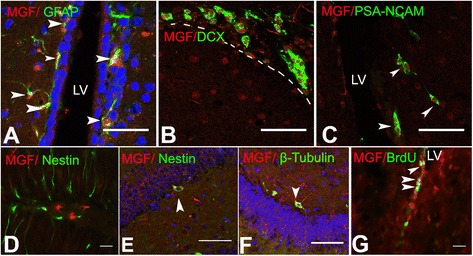
Characterization of endogenous MGF-expressing cells in the brain. **a** In the SVZ, MGF positive cells also express GFAP, which is a marker of neural stem cells in the SVZ. **b** Immature neurons in the SVZ were positive for both DCX and (**c**) PSA-NCAM expression and co-expressed MGF. These double positive neurons were in the RMS. **d** In the hippocampus, astrocyte-like MGF positive neurons did not express Nestin in the radial-glial like neural progenitors (type-1 NPCs) in DG area. **e** Nestin-positive amplifying neural progenitors in DG were positive for MGF in SGZ. **f** MGF was also expressed in the cytoplasm of β-Tubulin III positive neurons in the neurogenic region of DG. β-Tubulin III is one of the earliest markers seen in immature neurons. **g** BrdU-positive proliferating cells are also co-localizing with MGF in SVZ of LV in the brain. [*Red* is MGF, *Green* is GFAP (in **a**), DCX (in **b**), PSA-NCAM (in **c**), Nestin (in **e**), β-Tubulin III (in **f**) and BrdU (in **g**)]. (Scale bar (in **a**-**g**) 100 um)

To further identify what cell types express MGF, we performed serial immunofluorescence staining of MGF with multiple neural markers. Mice were given two injections of BrdU, 24 h apart and sacrificed 2 h after the last injection. Coronal sections were immunostained for neural progenitor cells (GFAP), immature neurons (DCX), migrating cells (PSA-NCAM), radial glia-like QNP cells (nestin) and microtubules (b-tubulin III). MGF co-localized with GFAP-positive cells at the SVZ of LV (Fig. 3a), indicating MGF is expressed in GFAP-positive cells with radial glial morphology that may represent quiescent neural stem cells of SVZ [4] that have neurogenic potential [27]. Of note, other adult brain regions did not show co-labeling with GFAP and MGF demonstrating that adult astrocytes do not express MGF (data not shown). Immunostaining showed co-localization of MGF with double cortin (DCX), a microtubule- associated protein that is expressed transiently in newly generated neuronal precursor cells and immature neurons only [28]. The MGF+/DCX+ double positive cells are clustered in the rostral migrating stream (RMS) (Fig. 3b) and the SVZ. In immature and migrating cells, MGF is highly expressed in the nucleus, surrounded by the DCX positive signal in the cytoplasm. This is similar to the co-localization of MGF+/PSA-NCAM+ we observed (Fig. 3c). In the dentate gyrus (DG) subarea of the hippocampus, radial glia-like QNP cells were positive for nestin staining. However, none of the cells demonstrated MGF co-localization (Fig. 3d). A small number of cells in the SGZ expressed MGF in the cytoplasm of nestin-positive cells (Fig. 3e) and were classified as the amplifying neural progenitors cells (ANP). These cells, also known as the transit amplifying population (TAP), are transitioning from the typical glial-like shape and extending processes toward the granular layers of the DG. ANP cells are derived from QNP cells through asymmetric division and are characterized by an oval-shaped cell body that runs horizontal to the SGZ and the absence of long processes like QNPs. Because of their distinct morphology and low numbers we did not specifically stain with markers for ANP/TAP cells [29, 30]. Additionally in the dentate gyrus, β-tubulin III positive neurons also show MGF expression in the cytoplasm (Fig. 3f). We found MGF was expressed robustly in immature neurons and may therefore be associated with neurogenesis. To determine if MGF was associated with proliferating cells, we co-stained for MGF and BrdU in BrdU injected mice. BrdU labeling is an established technique used to assess proliferation of a population of cells and to identify newly generated cells [31]. Proliferative progenitors cells were labeled with BrdU in S phase of the cell cycle at various developmental time points of the mouse. We found that BrdU-positive proliferating cells in the SVZ were also MGF positive and co-localized in the nucleus of cells (Fig. 3g). This suggests MGF is expressed in proliferating cells in the neurogenic niche and may contribute to neural development.

### MGF enhances neural progenitor proliferation and post-mitotic neural differentiation in the brain, but has no direct effect on neuron maturation

**Fig. 4:**
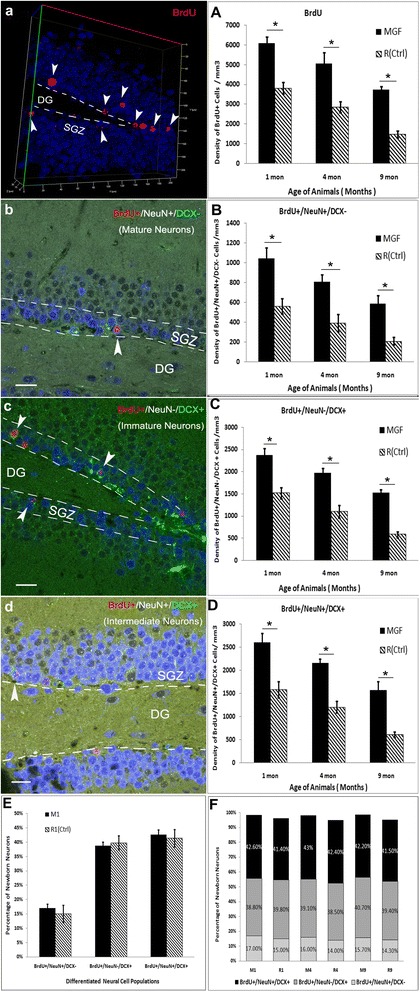
Regulation of MGF in the proliferation and maturation of newborn neurons in the adult hippocampus. **a** Representative Z stack/3D structure of the dentate gyrus (DG) of the hippocampus (40X) showing BrdU-positive proliferating cells in the subgranular zone (SGZ). (**a**) BrdU density between MGF transgenic mice and the age-matched control mice; MGF overexpression enhances the cell proliferation. **b** Representative image of new adult-born mature neurons in the SGZ labeled with BrdU+/NeuN+/DCX- . (**b**) MGF overexpression increases the density of new adult-born mature neurons, (**c**, **c**) new adult-born immature neurons labeled with BrdU+/NeuN−/DCX+. (**d**, **d**) differentiated intermediate neurons in transition from immature to mature neurons at 1,4 and 9-month-old,compared to all age-matched control mice. (**e**) At the age of 1 month about 15% of newborn neurons are mature, about 40% are immature and about 42% are overlapping between mature and immature. However, compared to the control, the MGF transgenic mice have no difference regarding the distribution of newborn neurons at different maturation stages. (**f**) The percentage of the newborn neurons at different maturation stages are very similar between MGF transgenic and control mice when they are aging. (*Error bars* indicate ± SEM. * *p* < 0.01, *N* = 4 each group); (scale bar 20um)

It is well known that adult neurogenesis in the brain undergoes four developmental stages, including proliferation, fate specification, migration and maturation/synaptic integration [32]. Hippocampus is a neurogenic region that has been extensively studied. To better understand how MGF is involved in neurogenesis we immunostained hippocampus from mice that constitutively overexpressed MGF (M mice) at different ages. Age matched R mice were used as controls. We injected adult mice at 1, 4 and 9 month old with BrdU (50 mg/kg body weight, i.p.) once daily for 7 days and examined the expression of BrdU 4 weeks after the first injection. Stereological quantification showed a significant increase in the BrdU+ proliferative cells (Fig. 4a) in hippocampal DG of adult brain in the MGF overexpressed brains compared to the controls (Fig. 4a; *p* < 0.01). The BrdU+ cell densities of MGF mice were about twice than that of the age-matched control mice of 1, 4 and 9 months old. This suggests that MGF can increase progenitor proliferation. Furthermore, BrdU density of control mice at 9 months old decreased to 39.03% ± 6.3% of the mice at 1 month of age. While, MGF mice at 9 months old had 61.4% ± 4.3% BrdU density of the MGF mice at 1 month old. This suggests that aging is the main factor contributing to impaired neurogenesis and MGF overexpression can significantly prevent the age-dependent decrease of progenitor cell proliferation. (*p* < 0.01)

Additionally, to determine whether the differences in neural progenitor proliferation lead to a net change in the number of newly generated mature (NeuN-positive) or immature (DCX-positive) neurons, we immunostained for these neural subpopulations in BrdU+ cells. Densities of both mature (BrdU+/NeuN+/DCX-) (Fig. 4b and B) and immature (BrdU+/NeuN−/DCX+) (Fig. 4c and C) neurons in the dentate gyrus of adult MGF overexpressing, M mice, was significantly higher than that of the age-matched control R mice (*p* < 0.01). This increase was also observed in intermediate neurons (BrdU+/NeuN+/DCX+) (Fig. 4d and D *p* < 0.01). Thus, MGF overexpression increased the total population of BrdU+, postmitotic neural stem cells during adult hippocampal neurogenesis.

To better understand at which stage in neurogenesis MGF was acting on, we examined the ratio of mature, immature and intermediate neurons to the total BrdU+ cell population. We compared the ratios between mice that began to conditionally overexpress MGF at 1 month and, mice that continually overexpressed MGF throughout life (M mice) and age-matched controls (R mice). We found no significant difference in the percent of mature, intermediate and immature neurons compared to the total BrdU+ cell population (Fig. 4e; *p* > 0.05). There was also no significant difference at 4 or 9 months of age (data not shown). The ratios of mature, immature and intermediate neurons had no significant difference between MGF overexpressing mice and age-matched controls in all age groups (Fig. 4f; *p* > 0.05). Because the total BrdU+ cell population increases but the ratio of postmitotic neuron subpopulations do not change, it suggests that MGF primarily affects the number of proliferative progenitor cells capable of becoming postmitotic neurons.

### Overexpression of MGF protects mice from age-related defects in olfactory function and contributes to increased olfactory neurogenesis in the aging brain

**Fig. 5:**
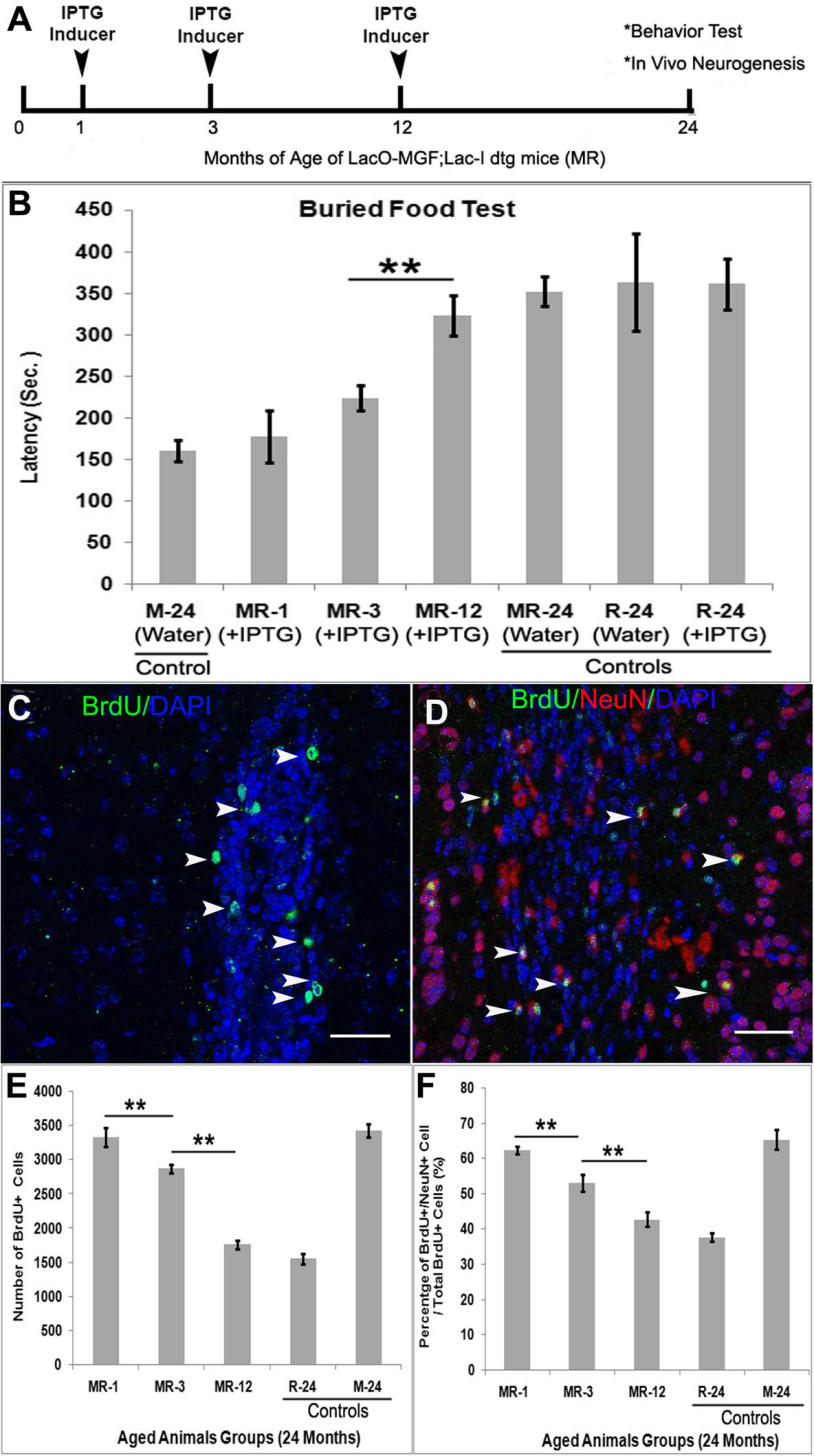
MGF overexpression preserved age-related Olfactory deficits and enhanced neural stem cell proliferation and differentiation. **a** Three groups of MR mice were separately fed with IPTG inducer in the daily water beginning at 1,3,12 month old (MR-1,MR-3,MR-12) and terminated at the age of 24 months old followed by function analysis and in vivo neurogenesis analysis. **b** Among all these 24-month-old mice, mice with constitutive overexpression of MGF throughout life (M-24) or induced overexpression of MGF from 1 month (MR-1) or 3 months (MR-3) of age had significantly improved olfactory function compared to the control mice of MR dtg mice (MR-24) with water (no IPTG) or MR dtg mice that began IPTG induction at 12 months (MR-12). MR-1, MR-3 and M-24 mice found buried food more rapidly. There was no difference among other control groups (*n* = 4–8 mice per group; bars represent mean ± SEM). The newborn neurons in olfactory bulb were quantitated after immunofluorescence stains. **c** Representative confocal images showing BrdU+ proliferating cells in the SVZ (**d**) differentiated mature granular neurons in olfactory bulb. **e** Quantification of total BrdU+ cells of olfactory bulb (**f**) and the percentage of newly generated granular neurons (BrdU+/NeuN+) from the total BrdU+ proliferating cells in olfactory bulb were quantitated. Early induced overexpression of MGF in the brain beginning at 1 month and 3 months had significantly enhanced the total number of BrdU+ proliferating cells and the average percentage of newly differentiated granular neurons (BrdU+/NeuN+) in the olfactory bulb by the age of 24 months. However, later overexpression of MGF induced at the age of 12 months had increased the percentage but not significantly compared to the control mice(R mice). *Green*(BrdU), *Red*(NeuN), *Blue*(Nuclear). (*Error bars* indicate ± SEM. **p < 0.01. Scale bar = 50 um

NSC migrate from the SVZ of the hippocampus to the OB where they functionally integrate into the neuronal network as granule interneurons [33–35]. Modulation of SVZ derived NSC integration into the local neural network parallels changes in the order discrimination test. Aging also decreases olfactory function causing a decline in the sense of smell [36]. For our study, we selected the buried food test for olfactory function and observed this behavior at different ages. MR mice from 1 (young), 3 (adult) and 12 (middle-aged) months of age referred to as MR1, MR3, and MR12, were induced by IPTG to conditionally overexpress MGF in the brain using the *lac*-operon system (Fig 5a). At 24 months old the animals were tested for olfactory function. To observe olfactory behavior a piece of cereal was buried in the bedding of a clean mouse cage. Using a stopwatch the mouse was timed for how long it took to find the food. Deficiencies in olfactory function are associated with time delays in finding the buried food (latency). MGF overexpression decreased the amount of time it took the animal to find the food at 1 and 3 months of age Fig. 5b. The buried food assay showed no significant difference in latency between MR-1 and MR-3 mice (*p* > 0.05). However, latency was significantly longer in the MR-12 group (*p* < 0.01) but was not significantly different from 24-month old control MR (MR-24) or control R mice (MR-24) +/− IPTG inducer (*p* > 0.05). M mice with constitutive expression of MGF throughout life showed the best performance at the age of 24 months old (M-24). This data indicates the earlier you induce MGF in development, the better preserved olfactory behavior is at 24 months of age and MGF has an age related effect. We also found similar results in preserving hippocampus-related spontaneous alternation by Y-maze test (*See* Additional file 1: Figure S1).

BrdU is incorporated in dividing cells and retained long-term in. This allows tracing of cell lineage, differentiation and survival. SVZ-derived progenitors migrate to the Olfactory Bulb. Extended BrdU administration allows newly generated, BrdU-labeled cells derived from SVZ stem cells to be tracked [23]. All subject animals at 24 months old were given BrdU water for 2 weeks. Neurogenesis was determined by counting the number of BrdU+ granule interneurons (BrdU+/NeuN+) in the granule cell layer of the OB. BrdU+ proliferating cells were seen in the SVZ neurogenic area (Fig.5c). These proliferating cells eventually migrated and reached the targeted region of OB where they differentiate into mature neurons (BrdU^+^/NeuN^+^) (Fig. 5d). The largest number of BrdU+ cells in OB among all groups was seen in the M group (M-24) (Fig. 5e), which had continuous overexpression of MGF in the brain throughout development and life. The number of BrdU+ neurons was twice that of R control animals (R-24), which had the least number of BrdU+ cells in the OB. When given IPTG inducer starting at 1 month of age to induce MGF overexpressed, MR mice (MR-1) had similar levels of BrdU+ proliferating cells to the continuous MGF overexpressing mice (M-24 as positive control). This suggests that induction of MGF overexpression at a young age, (1-month-old) prior to the age-related decline in neurogenesis in the SVZ, will prevent the age-dependent loss of neurons. When the MR mice were induced to overexpress MGF from adult age (3 months), the level of BrdU+ cells was significantly higher than the control group (R-24), (vs MR-1 group, **p < 0.01). However, if induction began at middle age (12 months) (MR-12), the number of BrdU+ cells was increased but not significantly compared to the R control group (R-24) (*p* > 0.05). This suggests that induction of MGF expression at middle age (12 months old), when significant neural loss (by 12 months) occurs, is unable to significantly restore neurogenesis and reverse neuronal loss in the brain. This paralleled the pattern of newly generated BrdU+ neurons. The average percent of BrdU+ mature granule neurons (BrdU^+^/NeuN^+^) showed the same trend (Fig. 5f). Both MR-1 and MR-3 groups have the highest portion of BrdU+ olfactory granule neurons at elder-age of 24 months, while the MR-12 group has no significantly increased level of BrdU+ mature neurons at 24 months.

### MGF is acting as a mitogen to promote neural proliferation in vitro

**Fig. 6:**
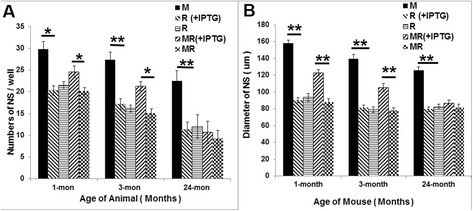
In vitro cell culture of SVZ-derived neurosphere (NS). Comparison of average NS numbers per well (**a**) and NS size at 7DIV (**b**). NSCs from M mice have significantly highest number and biggest size of NS at all age points (*p < 0.05,**p < 0.01), while NSC from R mice have no difference in neither sphere number nor the size (*p* > 0.05). IPTG induction in the MR mice-derived NSCs at age of 1 and 3 month, would significantly result in increasing of NS number and size (**p < 0.01)

Cultured Neurospheres (NS) were used to compare cell proliferation of SVZ-derived NSCs before and after induction of MGF expression and the mitogenic effect of MGF was measured. If MGF is a mitogen for neurogenic cells, then both the number of NS and NS diameter, which reflect the proliferation of both stem and progenitor cells, will be larger in NS treated with IPTG and smaller in untreated NS. We cultured NS from 1, 3 and 24 month old MR double transgenic mice and their aged-matched controls (M and R single transgenic mice). At 7 DIV, both the number and diameter of NS was observed in the presence or absence of IPTG. As shown in Fig. 6a, NSCs from continuous MGF expressing mice (M mice) showed the highest numbers of NS in all age groups. Although the average number of NS decline with age in the M group, NS numbers were significantly higher than other age-matched R and MR groups (± IPTG) (*p < 0.05, **p < 0.01). NSCs from R mice also had an age-dependent decrease of NS numbers but didn’t show obvious different of NS numbers with or without IPTG added in culture medium (*p* > 0.05). IPTG alone had no effect on the NSC. In the MR group, IPTG-treated NSC significantly demonstrated higher numbers of NS, compared to the aged-matched NSC with no IPTG treated at age of 1 and 3 month (*p* < 0.05). This difference did not occur at 24 months (*p* > 0.05). This indicates that the IPTG inducer turned on MGF overexpression and increased neural stem cells proliferation in young and adult aged mice, but not older aged mice. Similarly, NS size, as presented in Fig. 6b, M mice had the largest NS at 1, 3 and 24 months of age compared to the age-matched mice (R and MR mice) (**p < 0.01,*p < 0.05). IPTG itself in the R mice group didn’t significantly change the size of NS in any age group. However, it significantly increased the sphere size in the MR group at the age of 1 and 3 month (*p* < 0.01). No difference was identified at 24 months age (*p* > 0.05).

## Discussion

For the first time we have shown that MGF supports neurogenesis in the brain and this support is age dependent. Immunohistochemistry showed endogenous expression of MGF in the neurogenic regions of the brain and overexpression of MGF increased the number of neural stem cells in these regions. MGF also increased the number of neural stem cells in culture, indicating MGF is mitogenic. In vivo, MGF increased the number of neural progenitor cells, which correlated with improved olfactory behavior. However, the ability of MGF to affect the neural progenitor cell population was age dependent. The younger the animal was at the time of MGF up-regulation, the larger the increase in the neural stem cell populations, indicating MGF has an age related affect. This may be due to the age related decrease in the neural progenitor cell population.

We identified that MGF was expressed in areas of the brain associated with neurogenesis, which are the areas responsible for memory and emotion [1]. These areas included MGF-positive cells within the SGZ of the hippocampus, the ventral pallidum, the SVZ, the paraventricular (V3) region of the hypothalamus and the striatum. The SVZ is a known site of neurogenesis and self-renewing neurons in the adult brain [37]. It serves as a source of NSCs in the process of adult neurogenesis. It harbors the largest population of proliferating cells that will migrate through the rostral migrating stream (RMS) to reach the olfactory bulb and differentiate into interneurons. Our study shows that MGF is involved in neurogenesis by increasing the total population of neural stem cells in the hippocampus and olfactory bulb. The total number of BrdU+ cells increased with MGF but the ratio of the immature, intermediate and mature neurons did not change. This data indicates that MGF is increasing NSCs through proliferation or preservation of neural progenitor cells. This is also supported by our findings in vitro. MGF increased the number and size of the SVG-derived neural stem cells in culture. IGF-1 has been shown to increase neurogenesis through differentiation and maturation of neural progenitor cells in the hippocampus [38]. IGF-1 increased progenitor cell maturation and branching that was associated with improved special memory. Thus both MGF and IGF-1 may be involved in neurogenesis and prevent brain dysfunction. However, they likely act on different stages of the neurogenic process. The hippocampus is an area of the brain involved in epileptogenesis [39]. Ectopic neurogenesis might therefore have the unintended consequence of being epileptogenic. We did not observe any form of seizure like activity in any of the animals where MGF was over-expressed.

MGF is an alternative spice variant of IGF-1 and the two have very different functions. IGF-1 is a secreted protein and acts through its plasma membrane bound receptor (IGF-1R). IGF-1 induces cell proliferation, differentiation, migration and survival in neurogenesis [40–44]. MGF is an autocrine/paracrine peptide and its function is independent of the IGF-1R, suggesting that MGF acts through a different receptor or pathway [45]. MGF and IGF-1 mRNA are differentially regulated. In C2C12 (mouse muscle) cells, Growth Hormone (GH) stimulation increased MGF mRNA after 1 h of GH exposure followed by increased IGF-1 mRNA at 4 h [46]. This regulation of MGF and IGF-1 mRNA may play a role in GH activation of muscle satellite cells. This is also consistent with the hypothesis that MGF is acting on neural progenitor cell proliferation followed by differentiation and maturation initiated by IGF-1. MGF and IGF-1 were compared for their ability to rescue motor neurons in a facial nerve injury rat model [16]. MGF was able to preserve 88% of the motor neuron population while IGF-1 preserved only 37%. This indicates that MGF may be more effective than IGF-1 in protecting neurons.

The effect of MGF on neurogenesis is age dependent. When we looked at the number of BrdU+ cell in the brain of constitutively MGF overexpressing mice, we found the total number of BrdU+ cells decreased with age. MGF was able to increase the number of BrdU+ cells at 1, 4 and 9 months of age. However, there was a similar age related decrease as observed in controls. When we conditionally induced MGF expression we found increased numbers of BrdU+ cells in the olfactory bulb and improved olfactory behavior. Overexpression of MGF starting from 1 month resulted in significant improvements of both buried food olfactory function. The younger we induced the MGF expression the larger the number of BrdU+ cells and the better olfactory response was observed. Mice induced at 12 months of age were not significantly different from untreated controls. As the neural progenitor cell population becomes dysfunctional with age MGF is not able to induce mitogenic effects.

Neurogenesis in the adult vertebrate and human brain was first identified 25 years ago [47]. It has been almost exclusively studied [48] since then and it has become clear that dysregulation of adult neurogenesis is important in disease [49]. Enhancing adult neurogenesis is a therapeutic target for both age and disease related neuron loss. MGF is a small peptide of 25 amino acids. Direct delivery of MGF to the nervous system could target its effects and eliminate any unwanted side effects elsewhere in the body. However, it is important to understand that we do not understand the cellular site or mechanism of action of MGF. We have demonstrated that endogenous MGF is highly expressed in proliferating cells in the neurogenic niches, that expression declines with age and that maintaining youthful levels of expression preserves neurogenesis into old age. It is possible that MGF promotes neurogenesis by inhibiting cell death of new born neurons and future studies are directed towards understanding this mechanism. In these studies we have endogenously overexpressed MGF broadly in in neurons using the HD promoter. We therefore do not know whether it is active within the cell or by secreted, presumably receptor mediated, autocrine or paracrine effects. In the future, selectively expressing MGF only in neurogenic areas will allow us to understand whether the observed cellular and broader behavioral effects are autonomous to these cells or due to a broader influence on the brain involving overexpression in “non-neurogenic” areas. The possibility of maintaining youthful levels of neurogenesis throughout aging has tremendous therapeutic potential. Manipulation of MGF provides a very powerful tool for regulating this process. It is also to therapeutics that overexpression preserved youthful neurogenesis, but did not restore it when MGF was overexpressed in late adulthood. New therapeutic targets will be generated by understanding the mechanistic consequences of up and down-regulating MGF.

## Additional file

Additional file 1: Figure S1. Y-Maze spontaneous alternation test. (DOC 141 kb)
