# Supplementary material for: Mechano growth factor, a splice variant of IGF-1, promotes neurogenesis in the aging mouse brain
Source: Mol Brain. 2017 Jul 7;10:23. doi: 10.1186/s13041-017-0304-0 (PMC5501366; doi:10.1186/s13041-017-0304-0)
Supplement: Additional file 1: Figure S1. — Y-Maze spontaneous alternation test. (DOC 141 kb) [file 13041_2017_304_MOESM1_ESM.doc]

Appendix A. Additional file


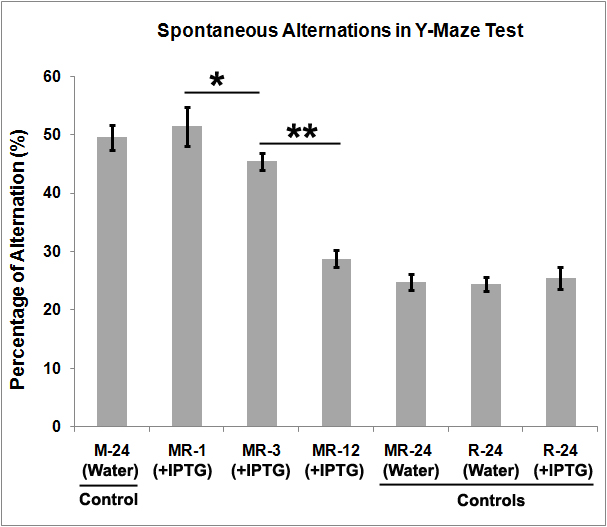


**Additional file 1 Figure S1. Y-Maze spontaneous alternation test.** It is to measure the willingness of rodents to explore new environments. The percentage of alternation was significantly reduced in control mice of both MR-24 and R-24, compared with mice with constitutive over-expression of MGF throughout life (M-24) or induced over-expression from 1 month of age (MR-1). There was a progressive decline in performance depending on the interval between birth and induction (MR-3, and MR-12) . (n=4-8 mice per group; bars represent mean ± SEM).
